# HU promotes higher order chromosome organization and influences DNA replication rates in *Streptococcus pneumoniae*

**DOI:** 10.1093/nar/gkaf312

**Published:** 2025-04-22

**Authors:** Maria-Vittoria Mazzuoli, Renske van Raaphorst, Louise S Martin, Florian P Bock, Agnès Thierry, Martial Marbouty, Barbora Waclawiková, Jasper Stinenbosch, Romain Koszul, Jan-Willem Veening

**Affiliations:** Department of Fundamental Microbiology, Faculty of Biology and Medicine, University of Lausanne, Biophore Building, Lausanne, CH-1015, Switzerland; Department of Molecular Microbiology, Groningen Institute of Biomolecular Sciences & Biotechnology, University of Groningen, 9747, The Netherlands; Department of Fundamental Microbiology, Faculty of Biology and Medicine, University of Lausanne, Biophore Building, Lausanne, CH-1015, Switzerland; Department of Fundamental Microbiology, Faculty of Biology and Medicine, University of Lausanne, Biophore Building, Lausanne, CH-1015, Switzerland; Institut Pasteur, CNRS UMR3525, Université Paris Cité, Unité Régulation Spatiale des Génomes, Paris, 75015, France; Institut Pasteur, CNRS UMR3525, Université Paris Cité, Unité Régulation Spatiale des Génomes, Paris, 75015, France; Department of Molecular Microbiology, Groningen Institute of Biomolecular Sciences & Biotechnology, University of Groningen, 9747, The Netherlands; Department of Fundamental Microbiology, Faculty of Biology and Medicine, University of Lausanne, Biophore Building, Lausanne, CH-1015, Switzerland; Institut Pasteur, CNRS UMR3525, Université Paris Cité, Unité Régulation Spatiale des Génomes, Paris, 75015, France; Department of Fundamental Microbiology, Faculty of Biology and Medicine, University of Lausanne, Biophore Building, Lausanne, CH-1015, Switzerland

## Abstract

Nucleoid-associated proteins (NAPs) are crucial for maintaining chromosomal compaction and architecture, and are actively involved in DNA replication, recombination, repair, and gene regulation. In *Streptococcus pneumoniae*, the role of the highly conserved NAP HU in chromosome conformation has not yet been investigated. Here, we use a multi-scale approach to explore HU’s role in chromosome conformation and segregation dynamics. By combining superresolution microscopy and whole-genome-binding analysis, we describe the nucleoid as a dynamic structure where HU binds transiently across the entire nucleoid, with a preference for the origin of replication over the terminus. Reducing cellular HU levels impacts nucleoid maintenance and disrupts nucleoid scaling with cell size, similar to the distortion caused by fluoroquinolones, supporting its requirement for maintaining DNA supercoiling. Furthermore, in cells lacking HU, the replication machinery is misplaced, preventing cells from initiating and proceeding with ongoing replication. Chromosome conformation capture coupled to deep sequencing (Hi-C) revealed that HU is required to maintain cohesion between the two chromosomal arms, similar to the structural maintenance of chromosome complex. Together, we show that by promoting long-range chromosome interactions and supporting the architecture of the domain encompassing the origin, HU is essential for chromosome integrity and the intimately related processes of replication and segregation.

## Graphical Abstract

**Graphical Abstract:**
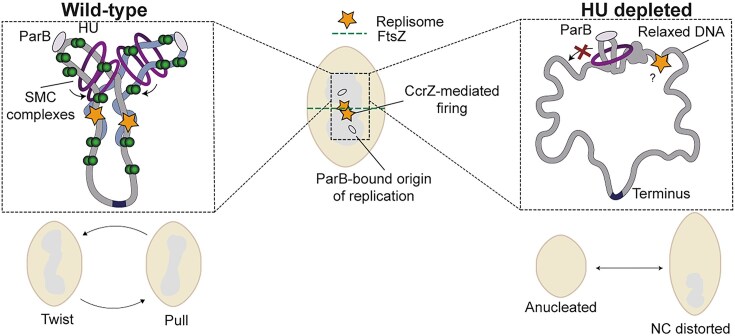

## Introduction

In bacteria, unlike eukaryotes, DNA replication, segregation and compaction occur simultaneously. Rather than a lack of organization, this points to an intimate connection and tight control of all these processes during the bacterial cell cycle. Chromosome compaction is mediated by several factors, including DNA supercoiling [1], transcription [2], protein complexes of the structural maintenance of chromosome (SMC) family [3], and nucleoid-associated proteins (NAPs) [4]. The degree of supercoiling and the abundance of NAPs and condensin complexes, along with their impact on chromosome structures, largely differ among bacteria [5]. Notably, the pneumococcus possesses the α subunit of the NAP HU (α-HU), which is highly expressed and crucial for growth [6, 7]. Furthermore, the global regulator MgaSpn shares DNA-binding properties with H-NS, suggesting that it may also act as a NAP [8]. Chromosome conformation capture coupled to deep sequencing (Hi-C) has provided detailed topological insights into the architecture of the genome within the cell of well-studied bacterial models [9–11]. Interestingly, the effect of HU loss on cell survival and large-scale structuring seems to differ widely between species [5]. Removing HU in *Escherichia coli* causes reduced viability, while it is lethal in *Bacillus subtilis* [12, 13]. Hi-C showed that *E. coli Δhupα/β* mutants are deficient in long-range DNA interactions and show an increase in short-range interactions [14]. Superresolution imaging of enlarged, circular *E. coli* confirms that large subdomains are substituted for smaller subdomains in *Δhupα/β* mutants [15]. In *Caulobacter crescentus*, removing HU promotes short-range contacts [16]. These findings suggest that the role of HU varies among species; however, the presence of multiple NAPs, their synergistic or antagonistic interactions, and their promiscuity in DNA-associated processes complicate the interpretation of the data. Other than its structural role, HU is directly involved in essential cellular processes, including replication [17], recombination and repair [18], and gene regulation [19–21]. Recently, HU was shown to be required for replication initiation in *B. subtilis* and *Staphylococcus aureus* [22–24] and for the organization of the origin of replication in *Mycobacterium tuberculosis* [25]. Pneumococcal HU was shown to be required to maintain DNA supercoiling [6], together with the DNA topoisomerase I (TopA) [26] and its regulator StaR [27].

Over the past decade, *Streptococcus pneumoniae* has become a key model organism for uncovering the molecular mechanisms that regulate the bacterial cell cycle. However, the involvement of HU in supporting different phases of the cell cycle has not yet been studied. In the pneumococcus, DNA replication occurs at mid-cell [28] and is orchestrated at the replication origin (*oriC*) by DnaA, whose activity is regulated by CcrZ [29]. Additionally, YabA negatively regulates initiation of DNA replication [30]. Besides the active process of DNA replication, another mechanism important for driving chromosome segregation is the *parS*/ParB system. ParB (partition protein B) binds to *parS* sites near the replication origin, recruiting SMC to ensure proper chromosome segregation [31]. RocS—a membrane-binding protein—interacts with ParB, localizes at the origin, and is required for segregation [32]. FtsK, which functions as a motor to pull through nearly segregated DNA in *E. coli* [33], might also be required for chromosome segregation in *S. pneumoniae* [7, 34]. Supercoiling and transcription also modulate chromosome segregation [35]. Surprisingly, most of the factors required for this process, such as ParB and SMC, can be deleted with only mild phenotypes [35, 36], suggesting that other factors or processes required for active segregation are yet to be elucidated.

Here, we employ a multi-scale approach of live-cell imaging and genome-scale analysis to dissect the role of HU in chromosome conformation, replication, and segregation. Tracking the pneumococcal nucleoid with HU using superresolution microscopy showed that it is extremely compact and dynamic. While HU binds transiently to the whole chromosome, chromatin immunoprecipitation-sequencing (ChIP-Seq) analysis revealed a binding preference in proximity of the origin of replication. By depleting HU, we observed a drastic loss of nucleoids, aberrations in cell size, and, consequently, a loss of scale between nucleoids and cell size. Quantification of DNA replication rates and integration of Hi-C data with fluorescence and superresolution microscopy provided a model in which HU-mediated structural (both short and large scale) and supercoiling defects are deleterious to the progression of the replication machinery and successful chromosome segregation.

## Materials and methods

### Bacterial strains and growth conditions

All strains, plasmids, and primers used are listed in Supplementary Tables S1 and S2. All pneumococcal strains in this study are derivatives of *S. pneumoniae* D39V and are listed in Supplementary Table S1. Construction of strains is described in the Supplementary material. Strains were grown in liquid C + Y medium at 37°C from a starting optical density (OD_600_) of 0.01 until the appropriate OD. Induction of the anhydrotetracycline (aTc)-inducible promoter (*Ptet*) was carried out by supplementing the medium with 25 ng/ml aTc (Sigma–Aldrich) and the isopropyl ß-D-1-thiogalactopyranoside (IPTG)-inducible promoter (*Plac*) with 1 mM IPTG (Sigma–Aldrich). Depletion strains were first grown in the presence of 25 ng/ml aTc up to OD_600_ = 0.1, then washed three times in fresh medium, diluted 15 times in fresh medium, and grown until the desired OD. Transformation of *S. pneumoniae* was performed as described previously [37] with cells taken at the exponential growth phase (OD_600_ = 0.1). When necessary, the medium was supplemented with the following antibiotics: chloramphenicol (4 μg/ml), erythromycin (0.5 μg/ml), kanamycin (250 μg/ml), spectinomycin (100 μg/ml), and tetracycline (0.5 μg/ml).

### Phase contrast and fluorescence microscopy

*Streptococcus pneumoniae* cells were grown in C + Y medium (pH 7.4) at 37°C to an OD_600_ = 0.1 with 25 ng/ml aTc at 37°C, after which they were washed three times with fresh C + Y medium, with or without the inducers when appropriate: IPTG for activation of dCas9 or complementation with fluorescent fusions (mCherry) or aTc for HU expression in depletion strain. A total of 1 ml of culture at OD_600_ = 0.1 was harvested by centrifugation for 1 min at 9000 × *g*. For DAPI staining, 1 μg/ml of DAPI (Sigma–Aldrich) was added to the cells and incubated for 2 min at room temperature before centrifugation. For imaging of exponentially growing cultures, cells were washed twice with 1 ml of ice-cold phosphate-buffered saline (PBS) and resuspended in 50 μl of ice-cold PBS. Briefly, 1 μl of cells was then spotted onto PBS pads. Pads were then placed inside a gene frame (Thermo Fisher Scientific) and sealed with a cover glass as described previously [38]. Microscopy acquisition was performed using either a Leica DMi8 microscope with an sCMOS DFC9000 (Leica) camera and a SOLA light engine (Lumencor) or a DV Elite microscope (GE Healthcare) with an sCMOS (PCO-edge) camera, a DV Trulight solid-state illumination module (GE Healthcare), and a ×100/1.40 oil-immersion objective. Phase-contrast images were acquired using transmission light (100-ms exposure). Fluorescence images were usually acquired with 700-ms exposure. The Leica DMi8 filters set used were as follows: DAPI (Leica 11533333, Ex: 395/25 nm, BS: LP 425 nm, Em: BP 460/50 nm), GFP (Ex: 470/40 nm Chroma ET470/40x, BS: LP 498 Leica 11536022, Em: 520/40 nm Chroma ET520/40 m), and mCherry (Chroma 49017, Ex: 560/40 nm, BS: LP 590 nm, Em: LP 590 nm). Images were processed using LasX v.3.4.2.18368 (Leica).

### SIM

Samples were prepared as for time-lapse wide-field microscopy, with the exception that for snapshots, the C + Y medium was substituted for PBS, and a high-precision 170-μm coverslip was used (VWR). 3D structured illumination microscopy (SIM) experiments were performed on a Deltavision OMX SR (GE Healthcare) microscope equipped with 488 nm and 568 nm lasers. 9–12 Z-stacks of 125-nm distance were acquired in 3D-SIM mode. For time-lapse imaging, a 3D stack was taken every 10 min, while a constant temperature of 37°C was maintained. Reconstruction and channel alignment were done in SoftworX (GE Healthcare) with ‘force modulation amplitude’ checked on and furthermore standard settings.

### PALM microscopy and analysis

Photoactivated localization microscopy (PALM) was performed on a Deltavision Elite microscope equipped with DLM (Deltavision Localization Microscopy), using a 60x oil 1.49 NA TIRF objective (Olympus), 405, 588, and 568 nm lasers, and an sCMOS camera. PALM imaging was done in Hi-Lo mode at 37°C. Cells were prepared as for wide-field time-lapse microscopy, where C + Y medium was substituted with PBS. Before each acquisition, a green fluorescence image (excitation: 488 nm laser, emission: 525/50 nm) and DIC image of the cells were made. After this, PALM images were acquired in the following sequence: activation (405 nm) 4× red excitation (568 nm), while red emission (632/60 nm) was recorded. The activation power was set to very low in the beginning of the acquisition and manually increased over time. Briefly, 5000 images were taken for each PALM acquisition.

### Particle tracking

To determine the mean square displacement (MSD) of HU-meos3.2, iSBatch (Caldas *et al.* [41]) was used to track HU-meos3.2 in the images acquired using PALM. For this, first, the regions containing the cells were selected in ImageJ, after which the peak fitter was used to find the fluorescent spots. Using the particle tracker, allowing for distances travelled not longer than 4 pixels/frame, only the spots appearing in multiple frames were kept. Tracks longer than 15 frames were removed. Finally, the collective MSD was calculated. This procedure was repeated for 10 PALM movies. All MSD tables were collected and plotted in R.

### Cell and nucleoid segmentation and analysis

Cells and nucleoids imaged using SIM were segmented using Morphometrics [39]. Cells and nucleoids imaged using phase-contrast/wide-field fluorescence microscopy were segmented using Oufti [40], where the nucleoids were segmented in object detection mode. DnaX foci were detected using the Peak Fitter tool from ISBatch [41]. To determine the cell and nucleoid dimensions in the PALM experiments, cell outlines were drawn by hand three times from DIC images (cells) and PALM reconstructions (nucleoids) using FIJI [42] and subsequently averaged. After segmentation, the wide-field and SIM microscopy data were analysed and visualized in R (https://cran.r-project.org/) using the R packages BactMAP [43] and ggplot2 [44]. To determine nucleoid size, dimensions, and intensity, first, the intensities from the TIFF values were added to the segmented nucleoids using BactMAP::extr_OriginalCells(). This returned the dimensions and size of the nucleoids and a list of pixel values per nucleoid. Of these values, the median and standard deviation were determined. The cell dimensions were imported from the Oufti or Morphometrics segmentations.

### Microtiter plate-based growth assay

For *S. pneumoniae* growth assays, cells were first grown in C + Y medium until the mid-exponential growth phase (OD_600_ = 0.3) with the inducer at 37°C, after which they were washed three times with fresh C + Y medium, with or without the inducers (IPTG or aTc) when appropriate. Cellular growth was then monitored every 10 min at 37°C in a microtiter plate reader (TECAN Infinite F200 Pro). Each growth assay was performed at least in triplicate. The average of the triplicate values was plotted, with the s.e.m. represented by an area around the curve.

### *ori/ter* ratio determined by whole-genome sequencing

Cells were pre-grown until OD_600_ = 0.3 in C + Y medium at 37°C in the presence of the inducer (aTc) for the HU depletion strain (VL5898) and in C + Y medium only for WT D39V. Cells were then washed three times with C + Y medium and diluted 15 times in 10 ml of fresh C + Y medium supplemented with or without the inducer and harvested for genomic DNA isolation after 1, 2, and 3 h of depletion (OD_600_ = 0.1–0.2). Genomic DNA was isolated using the FastPure Bacteria, DNA Isolation Mini Kit (Vazyme). Samples were sequenced with Illumina PE150 by Novogene, generating, on average 14 530 047 raw reads (maximum: 24 200 092; minimum 12 279 628). Raw Illumina sequencing reads were checked for quality using FastQC v0.11.8, available from https://www.bioinformatics.babraham.ac.uk/projects/fastqc/, and aligned to the D39V genome using Bowtie 2.0 [45]. Alignment files were used as input for index of replication (iRep) (available at https://github.com/christophertbrown/iRep) [46].

### Western blot analysis

Cells were grown in C + Y medium until OD_600_ = 0.1 and harvested by centrifugation at 8000 × *g* for 2 min at room temperature from 1 ml of culture. Cells were resuspended in 150 μl of Nuclei Lysis Solution (Promega) containing 0.05% sodium dodecyl sulfate (SDS), 0.025% deoxycholate, and 1% protease inhibitor cocktail (Sigma–Aldrich) and incubated at 37°C for 20 min and at 80°C for 5 min to lyse the cells. One volume of 4× SDS sample buffer [50 mM Tris–HCl (pH 6.8), 2% SDS, 10% glycerol, 1% β-mercaptoethanol, 12.5 mM ethylenediaminetetraacetic acid (EDTA), 0.02% bromophenol blue] was then added to three volumes of cell lysate sample and heated at 95°C for 10 min. Protein samples were separated by sodium dodecyl sulfate–polyacrylamide gelelectrophoresis (4–20%) and blotted onto polyvinylidene fluoride membranes (Merck Millipore). Membranes were blocked for 1 h with Tris-buffered saline (TBS) containing 0.1% Tween-20 (Sigma–Aldrich) and 5% dry milk and further incubated for 1 h with primary antibodies diluted in TBS, 0.1% Tween-20, and 5% dry milk. Commercial polyclonal rabbit anti-GFP IgG (Invitrogen A-6455) was used at 1:5000. Membranes were washed four times for 5 min in TBS and 0.1% Tween-20 and incubated for 1 h with secondary goat anti-rabbit IgG horseradish peroxidase conjugated (Abcam AB205718) diluted 1:20 000 in TBS, 0.1% Tween-20, and 5% dry milk. Membranes were then washed four times for 5 min in TBS and 0.1% Tween-20 and revealed with Immobilon Western HRP Substrate (Merck Millipore).

### ChIP-Seq and analysis

Cells were grown in 2 ml of C + Y, 37°C up to OD_600_ ∼ 0.4, diluted 50 times in 30 ml of preheated C + Y (37°C), and allowed to grow up to an OD_600_ of 0.2. The cells were harvested and fixated in 1% formaldehyde. The cells were washed several times in ice-cold PBS, and the pellets were snap-frozen and stored in −80°C. Subsequently, the cells were thawed and sheared in a Covaris S2 sonicator using the following protocol: 200 cycles, 100 W, 10% load, 12 min. A fraction of the cell mixture was taken apart (input fraction). Polyclonal anti-GFP (ThermoFisher A-6455) was bound to magnetic beads (Dynabeads Protein G 10004D). The remaining sheared cells (IP fraction) were added to the beads, and the mixture was incubated for 2 h on a rotating wheel for 2 h. After immunoprecipitation, the beads were washed. Cross-linking was reversed in both the input and IP fractions by shaking at 1400 rpm overnight at 65°C in shearing/chromatin buffer (SCS) containing 1% SDS, 10 mM EDTA, and 50 mM Tris (pH 8). DNA was purified using phenol–chloroform extraction followed by a polymerase chain reaction (PCR) purification kit (Qiagen QIAquick). Libraries were prepared by the Lausanne Genomic Technologies Facility (LGTF) using an Ovation Ultralow V2-DNA-Seq library preparation kit (NuGEN), and 100 nt paired-end (PE) sequencing was done at the LGTF on an Illumina HiSeq. Next to input and IP samples, wild-type D39V without a GFP-tagged protein was also processed and used as a mock for further analysis. For the analysis, sequence trimming, alignment, and peak annotation were done using a Snakemake pipeline (https://github.com/veeninglab/chip-seq). Sequence files were gunzipped, and sequence quality was assessed using FastQC. Data were trimmed using Trimmomatic [47], and sequences were aligned to the reference genome (D39V, GenBank CP027540) using Bowtie 2.0 [45], converted to *.bam* files using SamTools [48], and filtered to remove false positive peaks due to duplicated regions in the chromosome using Sambamba [49]. Genome mappability of D39V was analysed using umap [50], and regions with low mappability were blacklisted using bedtools [51]. Subsequently, peaks were annotated using MACS2 [52]. Relative sequence reads were normalized per sample, and enrichment of IP compared with mock was calculated and binned per 1000 bp for visualization using deeptools [53]. The resulting bedgraphs were plotted in R. The relative enrichment of HU per binned region (1000 bps) was plotted against RpoB enrichment and GC content (%) per 1000 base pairs.

### Hi-C procedure and sequencing

Cells were pre-grown until OD_600_ = 0.3 in C + Y medium at 37°C in the presence of the inducer (aTc) for HU-depletion strain (VL5898). Experiments were performed in duplicates, except for the Δ*smc* strain that was performed once. Cells were then washed three times with CY and diluted 15 times in 10 ml of fresh C + Y medium supplemented with or without the inducer and let grow for 3 h up to OD_600_ = 0.1–0.2. Cell fixation was performed with 3% formaldehyde (Sigma–Aldrich, cat. no. F8775). Quenching of formaldehyde with 300 mM glycine was performed at room temperature for 20 min under gentle agitation. The samples were recovered by centrifugation (4000 × *g*, 10 min, 4°C), washed with 10 ml 1× PBS, re-centrifuged, and stored at − 80°C until processing. Hi-C experiments were performed as described in [54], as well as DNA extraction, purification, and processing into a sequencing library. Proximity ligation libraries were sequenced using the PE Illumina sequencing (2  ×  35 bp, NextSeq500 apparatus) at the sequencing platform of the Institut Pasteur of Paris.

### Processing of reads and Hi-C data analysis

Reads were aligned to the reference genome with Bowtie 2.0 and Hi-C contact maps were generated using hicstuff v3.1.5 [55], employing default settings and DpnII and HinfI enzymes for digestion. Contacts were filtered according to the method described in [56], and PCR duplicates, identified as paired reads mapping to the exact same position, were removed. The resulting matrices were binned at intervals of 1, 5, 10, and 20 kb. Balanced normalization was achieved using the ICE algorithm [57], and the contact maps were stored in cool file format via cooler (version 0.8.11) [58]. Comparative analyses were conducted by binning matrices at 5-kb resolution, downsampling to equal contact numbers, and comparing using the log2 ratio.

## Results

### The pneumococcal nucleoid and HU are highly dynamic

**Figure 1.**
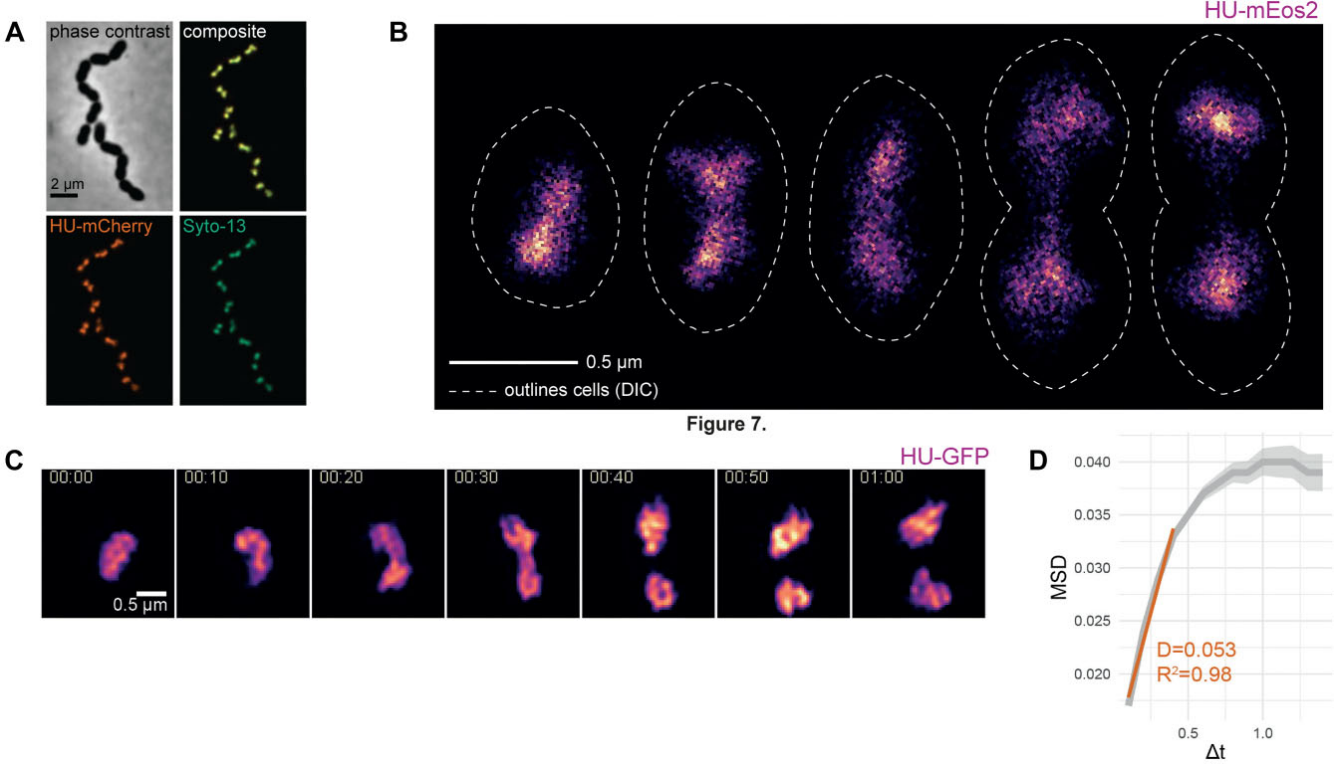
A dynamic, compact nucleoid robustly scales with cell size. (**A**) Deconvolved epi-fluorescence microscopy images of HU–sfGFP and the chromosome stained with DAPI show a near-perfect overlap. (**B**) Representative images of PALM microscopy reconstructions of 5 cells with increasing cell sizes. PALM reconstructions of experiment detecting HU-mEos3.2. Scale bar: 0.5 μm. Dashed lines: cell outline, drawn by hand from DIC images. (**C**) Time-lapse sequence of HU–sfGFP imaged using SIM, mid-section of 3D-SIM experiment. Images taken every 10 min, scale bar 0.5 micron. (**D**) MDS over time of HU-meos3.2. HU-meos3.2 recorded using PALM, particles were tracked, and collective MSD was calculated using iSBatch [41]. Apparent diffusion coefficient for the short time intervals was calculated by determining the linear slope between 0 and 0.5 s. Shade = standard error.

Because of its very high abundance and limited cellular diffusion, fluorescent protein fusions to HU are widely used to track live pneumococci in animal and tissue models as well as flow cytometry studies [59–62]. In other bacteria, HU has widely been used as a tool for live-cell tracking and measurement of nucleoid dimensions and dynamics [63–65]. To test whether *S. pneumoniae* HU is an appropriate proxy for the pneumococcal nucleoid, we performed co-localization studies using HU-FP fusions and DAPI. As shown in Fig. 1A, we observed near-perfect overlaps demonstrating that HU is a suitable marker for the pneumococcal nucleoid. This enables us to investigate the shape and dynamics of the nucleoid during the cell cycle in live *S. pneumoniae* cells.

To analyse the pneumococcal nucleoid in high temporal and spatial resolution in live cells, we performed time-lapse SIM of cells carrying a functional fusion of HU to superfolder GFP (HU–sfGFP) (Supplementary Fig. S1A). This showed that pneumococcal nucleoids are very dynamic, changing shape and structure over time (Fig. 1B). The bulk nucleoid splits at the end of the cell cycle (40 min), which is confirmed by the observation that 96% of single pneumococcal cells carry one bulk nucleoid (Supplementary Fig. S1B and C).

While SIM provides higher spatial resolution compared with wide-field epifluorescence microscopy, since the pneumococcal nucleoid has a diameter of ∼0.5 μm, we are imaging close to the resolution limit. To obtain deeper insights into the pneumococcal nucleoid shape in live cells, we set up PALM for *S. pneumoniae*. To perform PALM, we first constructed *S. pneumoniae* carrying HU fused to a codon-optimized photoswitchable mEOS3.2 [66]. In total, 160 pneumococcal cells carrying hu-meos3.2 were imaged, with a Nyquist resolution of 18 nm and an average number of 3990 ± 2730 events per cell. Cells were divided in five groups based on cell length. Consistent with the observations of the SIM, bulk nucleoids stretch out to the new mid-cell regions early in the cell cycle, but only the largest cells carry two separated nucleoids. Interestingly, in the second-largest cell group, the region between the dense domains of the nucleoid became highly stretched, measuring only 163 ± 45 nm, which is beyond the resolution limits of our SIM microscope. Not only is the nucleoid very dynamic, as shown by SIM (Fig. 1C and D), PALM shows that HU itself is also dynamically moving within the nucleoid space with a *D*_app_ of 5.3 × 10^–2^ μm^2^/s measured over short time intervals (<0.5 s). This is >20-fold more mobile than ParB_L.lactis_–GFP bound to a single *parS_lactis_* site at the origin of replication, measured previously using TIRF microscopy, and 10-fold more mobile than DnaX–GFP, however, more confined than free diffusing GFP [67], indicating that HU is transiently binding the nucleoid.

### HU displays low-intensity binding with bias towards the origin of replication

**Figure 2.**
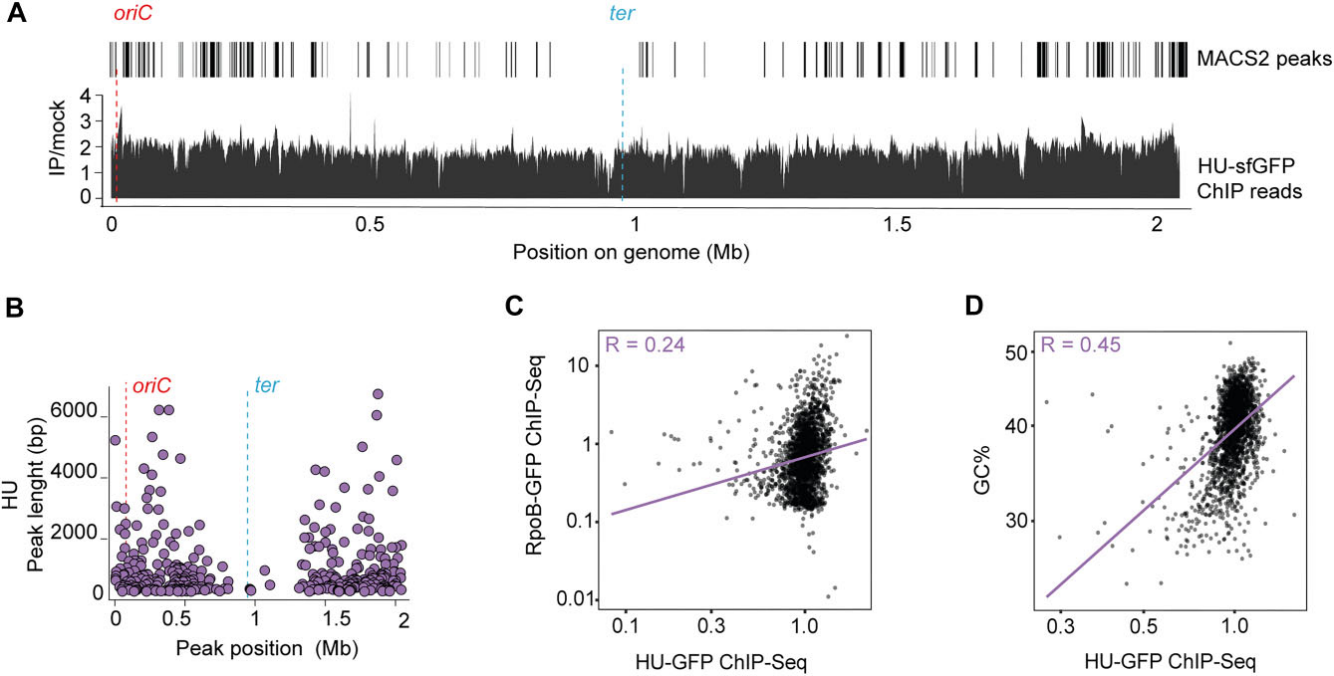
HU-binding profile and correlation with active transcription and GC content. (**A**) HU-binding profile obtained by ChIP-Seq. Normalized counts (IP/mock) binned per 100 base pairs obtained for the immunoprecipitated HU–sfGFP sample over the mock (wild-type) sample are plotted against the chromosomal coordinates. Significant (q-value < 0.05) peaks detected by MACS2 are depicted. Position of origin and terminus of replication are indicated on top of the graph. Note the asymmetric distribution of the left and right arms of the chromosome due to a chromosomal inversion in strain D39V. (**B**) HU occupancy peak size per position on the chromosome. (**C**) RpoB version HU occupancy (IP/mock reads per 1000 base pairs normalized with total reads). Pearson’s R = 0.24. (**D**) HU occupancy (IP/mock reads per 1000 base pairs normalized with total reads) versus GC content (%).

To assess the properties of HU binding at a whole-genome scale, we performed ChIP-Seq of pneumococcal cells carrying HU–sfGFP. Two independent cell cultures of exponentially growing bacteria were used for immunoprecipitation with an anti-GFP antibody, and wild-type (mock) samples were used as controls. After sequencing and bioinformatic analysis, we detected 132 reproducible high-confidence binding sites (q-value < 0.05) of enrichment along the entire chromosome, which are absent in the mock control (Fig. 2A). These peaks are widespread and have a relatively weak signal (log_2_ fold changes between 1 and 2), similarly to what has been observed for *E. coli* α/b HU subunits [68]. Interestingly, we detected only a few binding sites (5 out of 132) near the replication terminus (*ter*) (1–1.04 Mbp), indicating a bias in HU-binding distribution along the chromosome, with a preference for the origin *(ori)* region (Fig. 2B and Supplementary Table S3). Since ChIP-Seq sequencing data were adjusted using input DNA from cells in the same growth stage, we can exclude that this effect is due to the impact of replication-associated gene dosage effects typically seen during exponential growth. Additionally, peak lengths are variable and can reach up to 7 kb, indicative of a dispersed HU binding. Despite the relatively low enrichment levels measured that may be caused because of its dynamic ON/OFF binding (Fig. 1D), the high reproducibility of the distribution patterns and their detection with a strict statistical threshold (using q-value instead of *P*-value) resulted in binding profiles that are representative of HU.

Transcription imposes structural constraints on chromosome folding and could therefore affect HU-binding pattern. To test whether there is correlation between HU-binding sites and active transcription, we performed ChIP-Seq on cells carrying a functional GFP-marked β-subunit of RNA polymerase (RpoB–sfGFP) (Supplementary Fig. S2A and Supplementary Table S3). Magnification of highly transcribed genes such as the capsule operon *cps* and the ribosomal gene *rpsL* showed a high median RpoB occupancy at these chromosomal locations (Supplementary Fig. S2B). However, as shown in Fig. 2C, there was no clear correlation between RpoB and HU occupancy. Although HU homologs have been reported to prefer to bind to AT-rich regions, we observed a moderate correlation (Pearson’s R = 0.45) between HU-binding sites and GC-rich regions in the pneumococcal genome (Supplementary Fig. S2). Taken together, ChIP-Seq analysis showed that HU binds with low intensity to the whole chromosome, with a bias towards the origin of replication and GC-rich loci.

### Depletion of HU severely impacts growth and cell morphology

**Figure 3.**
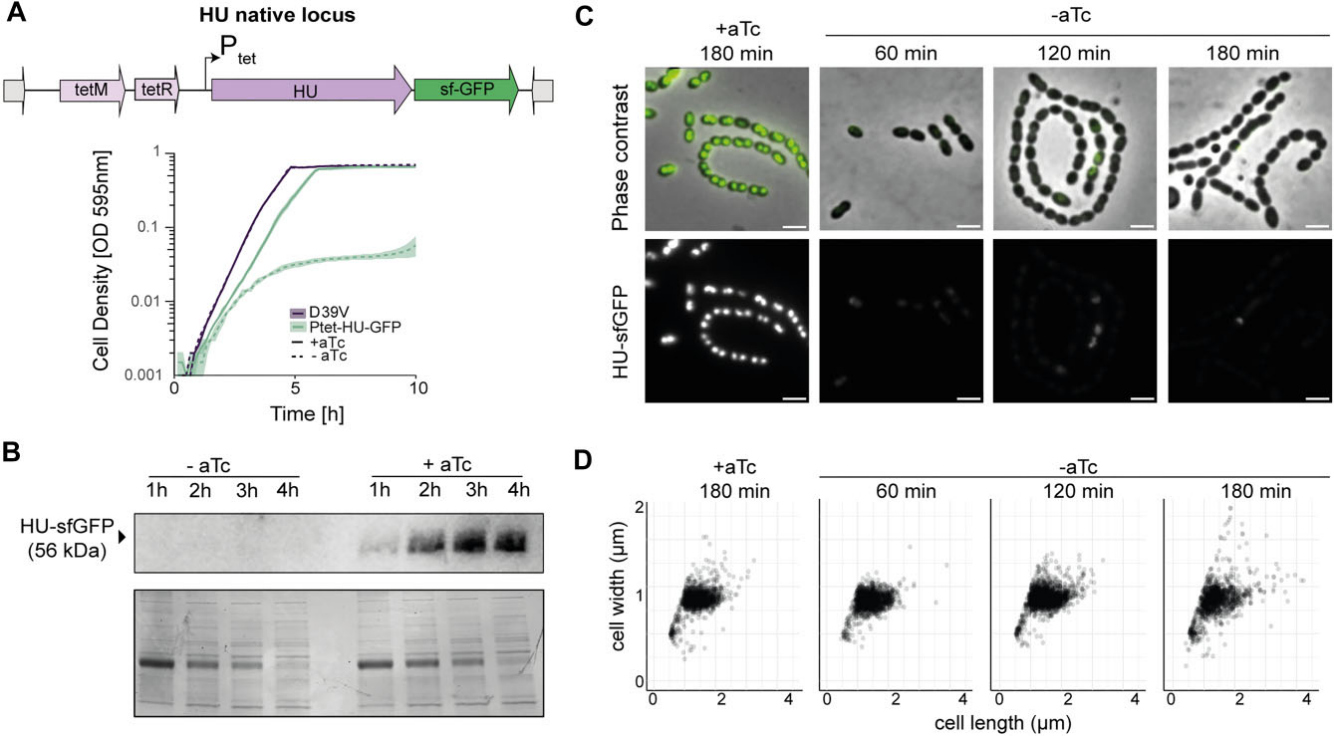
HU depletion kinetics and effects on cell morphology. (**A**) Overview of HU depletion strategy. The native HU promoter *Phu* was replaced with a *Ptet* promoter at the native locus. A selection marker (*tetM*) was added upstream, as well as *tetR* for aTc induction. Growth curves of cells with HU depletion (Ptet–HU–sfGFP) (−aTc) indicate a strong growth defect. (**B**) Depletion kinetics of Ptet–HU–sfGFP by immunoblot (anti-GFP antibody) at one, two, three and four h of depletion. Coomassie blue staining was used as a loading control (bottom). (**C**) Depletion kinetics of Ptet–HU–sfGFP by fluorescence microscopy. HU–sfGFP expression is drastically reduced after 1 h of depletion. Top: composite of HU–sfGFP and phase-contrast. Bottom: HU–sfGFP. Scale bar: 2 μm. (**D**) Distribution of cell width and length of HU-depleted cells.

To be able to tightly control HU expression and investigate its role in chromosome dynamics, we replaced the native HU promoter with an aTc-inducible promoter. Growing the cells in the absence of aTc led to a growth rate defect compared with the wild type and cells grown in the presence of the inducer (+aTc) (Supplementary Fig. S3A). Phase-contrast and fluorescence microscopy of cells stained with DAPI revealed defects in cell morphology and the appearance of a-nucleated cells (Supplementary Fig. S3B). To follow depletion kinetics, we fused Ptet–HU to sfGFP (Ptet–HU–sfGFP), which led to a drastic reduction of growth rate in the absence of the inducer (Fig. 3A). Even in the presence of the inducer, the depletion strain showed a growth defect compared with the wild-type D39V. To confirm that the growth defect observed in the absence of the inducer was solely due to reduced HU levels (p-HU, for pneumococcal HU), we complemented HU with a second copy of *E. coli α-*HU (e-HU), expressed ectopically under an IPTG-inducible promoter. e-HU was able to restore growth, as well as restore the loss of nuceloids (Supplementary Fig. S3C and D). Western blot analysis using anti-GFP antibodies showed that depletion of HU was very efficient with significantly reduced HU–sfGFP after 1 h and with a complete loss of signal after 3 h in the absence of inducer (Fig. 3B). Using fluorescence microscopy, we observed strains grown with and without aTc at three distinct time intervals to assess depletion kinetics and cell shape (Fig. 3C and D). Ptet–HU–sfGFP cells grown with aTc gave a bright signal that well distinguishes the pneumococcal nucleoid, which is lost after 1 h of depletion, confirming the immunoblot observations (Fig. 3B). While there was no significant difference in the total cell size distributions (Kolmorogov–Smirnov test, *P*< .05 for all combinations), a typical, severe morphological defect was visible, hallmarked by an increased irregularity in cell size within cell chains (Fig. 3C) and an increase in the percentage of extremely long cells [Fig. 3D, cell length > (mean + 2*sd)_+aTc_, +aTc = 2.9%, −aTc: 60 min = 13%, 120 min = 8.7%, 180 min = 12%]. These observations defined HU as an essential NAP, required for pneumococcal growth and cell shape in line with previous observations [6].

### Reducing HU levels alters nucleoid scaling dynamics

**Figure 4.**
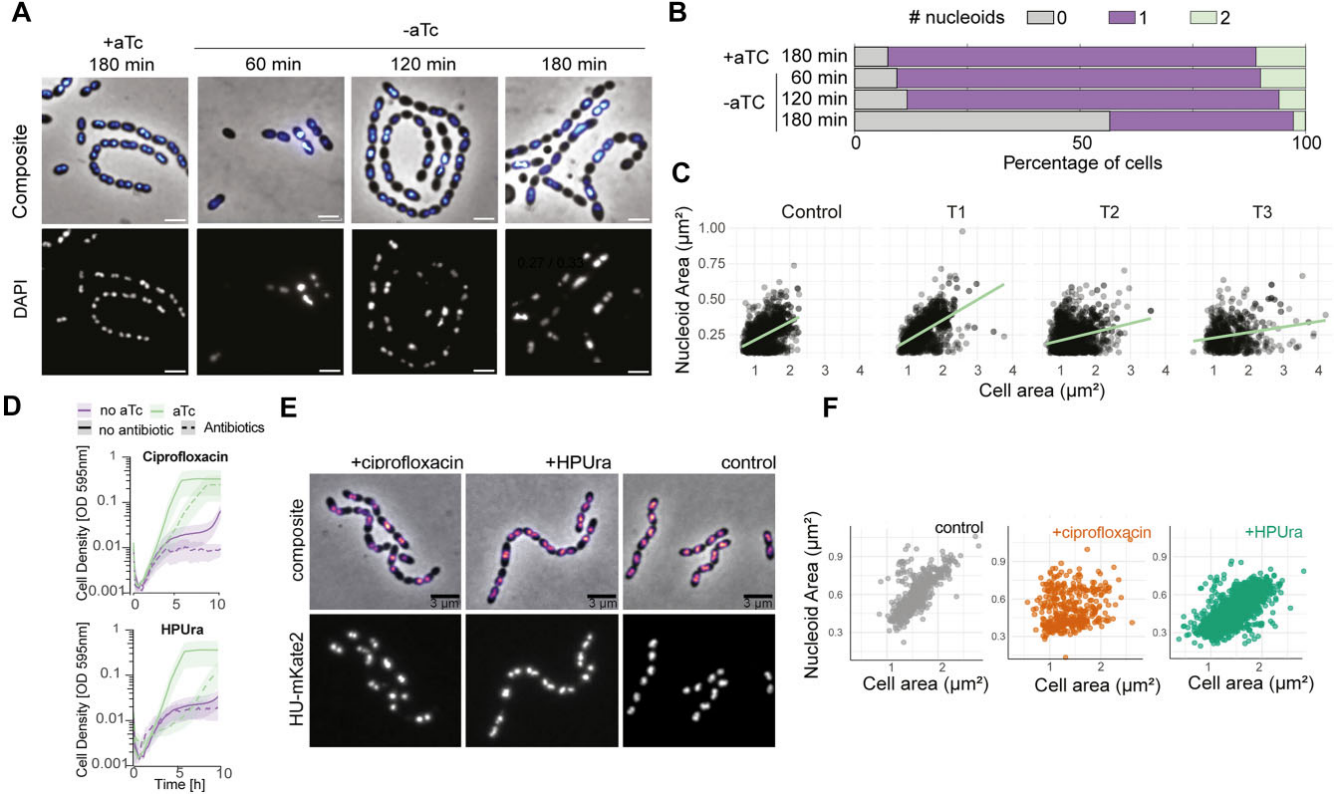
Depletion of HU strongly affects chromosome maintenance and NC scaling. (**A**) Fluorescence microscopy of HU-depleted cells stained with DAPI and imaged immediately afterwards. Scale bar: 2 μm. Cell area and nucleoid area were segmented from phase-contrast (cells) and fluorescence (nucleoids) with Oufti. (**B**) Percentage of cells lacking a nucleoid increases with time (55% after 3 h of depletion). (**C**) Nucleoid to cell area (NC) for HU-depletion strain grown in the presence (Control) and absence of aTc for 1 (T1), 2 (T2), and 3 h (T3). (**D**) Growth of HU-depletion strain in presence and absence of the inducer and with sub-MIC concentrations of HPUra (0.1 μg/ml) and CIP (0.2 μg/ml) E. Representative images of cells carrying *hu::hu-mKate2* grown with sub-MIC concentrations of CIP or HPUra, scale bar: 3 μm. (**F**) NC ratios are distorted in cells treated with CIP but not in cells treated with HPUra.

We next investigated nucleoid morphology upon HU depletion using wide-field fluorescence microscopy of cells stained with DAPI (Fig. 4A). Over time, in the absence of aTc, the percentage of anucleate cells increased, being 10% at 1 h (125 out of 1463 cells), 12.5% at 2 h (250 out of 2268 cells), and 55% at 3 h (851 out of 1547 cells) of depletion, suggesting a chromosome replication or segregation defect (Fig. 4B). The disruption of the chromosome integrity observed in an HU depletion strain was phenocopied by treatment with antibiotics affecting the cellular supercoiling state and DNA replication, as well as an *smc* deletion (Fig. 4E and F). Indeed, cells depleted of HU were sensitized to treatment with sub-inhibitory doses (sub-MIC) of antibiotics that inhibit DNA gyrase and topoisomerase IV (ciprofloxacin, CIP) and DNA replication (6-p-hydroxyphenylazo-uracil, HPUra) [69]. However, treatment with low doses of spectinomycin, which inhibits protein synthesis, did not show a synergistic effect with HU depletion (Supplementary Fig. S4).

In replicating wild-type pneumococci, the nucleoid is remarkably compact and robustly scales with cell size during growth, with a median nucleoid size of 0.5 μm^2^ and an NC ratio of 0.4. [Fig. 4C (first panel) and F (control)]. After three h of HU depletion, the NC ratio of cells that still have a nucleoid is drastically perturbed, with the appearance of larger cells with small nucleoids (Fig. 4C, Pearson’s R/median NC ratio: +aTc = 0.49/0.39, −aTc; 60 min = 0.57 / 0.37, 120 min = 0.27 / 0.33, 180 min = 0.21 / 0.32). To investigate what causes this NC perturbation, we measured the nucleoid size using HU-mKate2 as a nucleoid marker in cells treated with HPUra and CIP. Blocking DNA replication using sub-MIC concentrations of HPUra led to anucleate cells; however, the nucleoid scaling ratio stayed intact, confirming that NC scaling is independent of DNA replication (Fig. 4D–F) [70]. Cells lacking *smc* showed a phenotype very similar to HPUra treatment. Interestingly, when cells were grown in the presence of sub-MIC concentrations of CIP, the relationship of the nucleoid to cell size is lost due to aberrant cell and nucleoid sizes (Fig. 4D–F). Overall, we showed that depletion of HU severely impacts chromosome maintenance and disrupts the robust nucleoid scaling with cell size. Our data suggests that these phenotypes may be attributed to significant defects in chromosome transitions, including DNA supercoiling and DNA repair, resulting from HU depletion.

### HU promotes the higher organization of the pneumococcal chromosome

**Figure 5.**
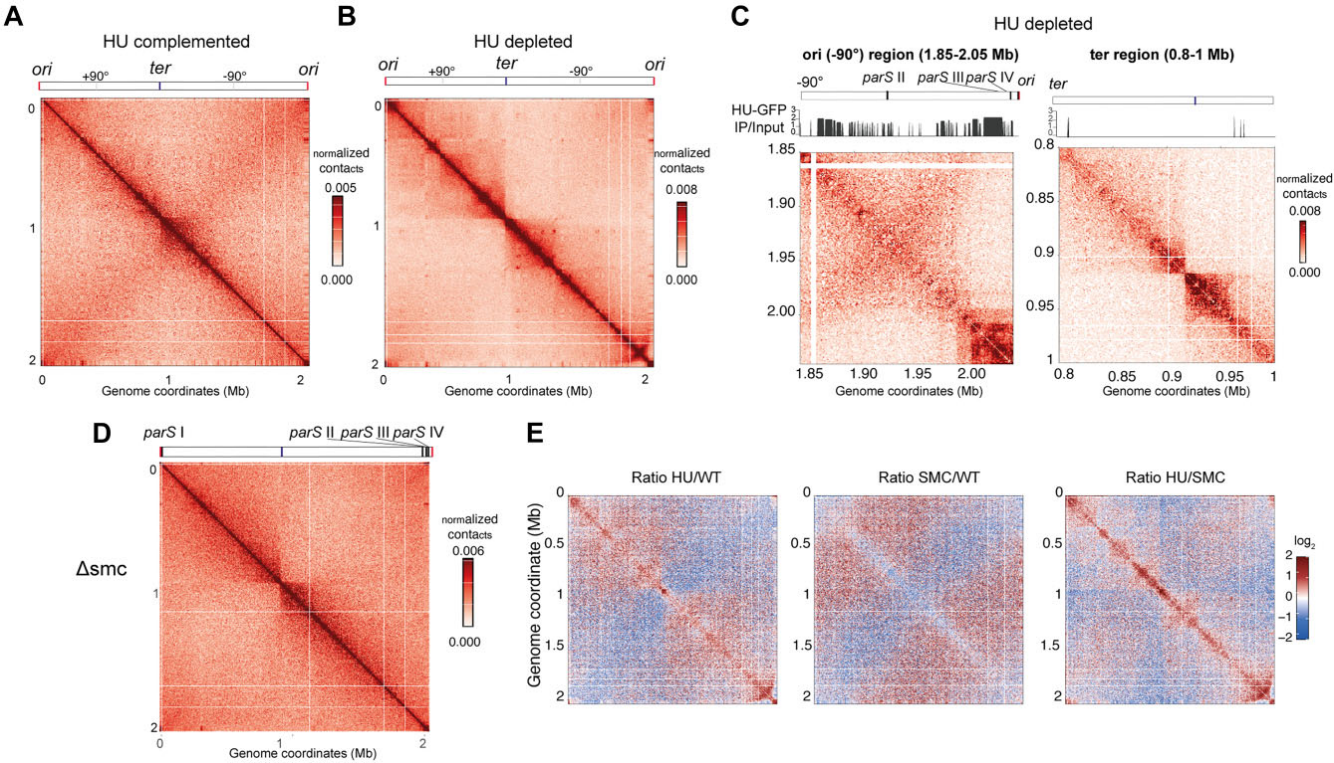
HU and SMC promote long-range chromosome folding. (**A**) Normalized Hi-C contact maps of HU-complemented cells (+aTc) at 5-kb resolution. Position of the origin and terminus of replication are depicted. (**B**) Normalized Hi-C contact maps of HU-depletion strain grown in the absence of aTc (3 h depletion) at 5-kb resolution. (**C**) Magnification of the origin on the left replichore (left panel) and terminus regions (right panel). *Ori*-, *ter*-, and *parS*-binding sites are depicted. (**D**) Normalized map of Δ*smc* at 5-kb resolution. (**E**) Differential contact maps corresponding to the log_2_ ratio of Hi-C interactions between WT and cells lacking HU or SMC and between cells lacking HU and SMC. The blue/red colour scale reflects the enrichment in contacts in one population with respect to the other.

To investigate the exact 3D organization of the pneumococcal nucleoid and the impact of HU in chromosome conformation, we performed Hi-C in HU-depletion strain. Cells were grown with and without aTc for three h until the exponential phase was reached (Fig. 5A and B). Samples were processed using Hi-C, and DNA was sequenced and analysed to produce contact maps. In cells grown in the presence of aTc and expressing HU (Fig. 5A), the contact map revealed, besides the expected diagonal signal resulting from contacts between neighbouring loci, a faint secondary diagonal. This secondary diagonal is a common feature of bacterial genomic Hi-C maps and reflects enrichments in contacts between chromosomal arms that are mediated by condensins [71–75]. HU-complemented cells lack structural signatures such as self-interacting or large domains, as observed for exponentially growing wild-type cells (Supplementary Fig. S5A). In the absence of HU, the normalized contact maps showed significant differences compared with WT conditions. First, contact matrices showed a reduction in long-range contacts (1 Mb) compared with the wild-type strain, concomitant with an increase in short-range contacts along the chromosome (Fig. 5A and B and Supplementary Fig. S5B). Secondly, replichores’ trans contact were strongly attenuated, with the terminus of replication clearly partitioning the chromosome. Interestingly, local structures also emerged in the absence of HU, including dots in the maps likely suggesting the presence of DNA loops, as well as a DNA stripe or ori-proximal ‘hairpin’ structure on the left replichore, of ∼100 kb (Fig. 5B and C). Magnification of 100 kb surrounding the *ori* on the left replichore (Fig. 5C) underlined a dense HU binding in this region, overlapping the *parS*-SMC loading sites II in correspondence with the hairpin and III and IV closer to the *oriC* [31]. At *ter*, the conformational signature reflects the increased *cis-*contacts between the two replichores due to loss of cohesion (Fig. 5C). To compare the impact of HU depletion with the absence of the SMC condensin known in other species to be responsible for replichore bridging, we performed Hi-C in a Δ*smc* mutant (Fig. 5D). As expected, the Hi-C contact map confirmed a role of SMC in bridging the pneumococcal chromosome replichores. Remarkably, the reduction of long-range contacts obtained in the absence of SMC is similar to what was observed in the absence of HU (Fig. 5D and Supplementary Fig. S5B). The ratio of normalized contact maps between wild-type, HU-depleted and Δ*smc* cells nevertheless pointed at differences between HU- and SMC-depleted chromosomes (Fig. 5E). Indeed, whereas HU depletion led to changes in the local folding of chromosomes, the loss of SMC had little impact on that organization. In particular, the segmentation at the level of the *ter* and, overall, the enrichment in short-range contact was less pronounced in the absence of *smc*, and the *ori-*proximal hairpin was absent. Altogether, these findings support a major role of HU in promoting long-range contacts and maintaining the conformation of the origin domain.

### HU is required for replication initiation and replisome localization

**Figure 6.**
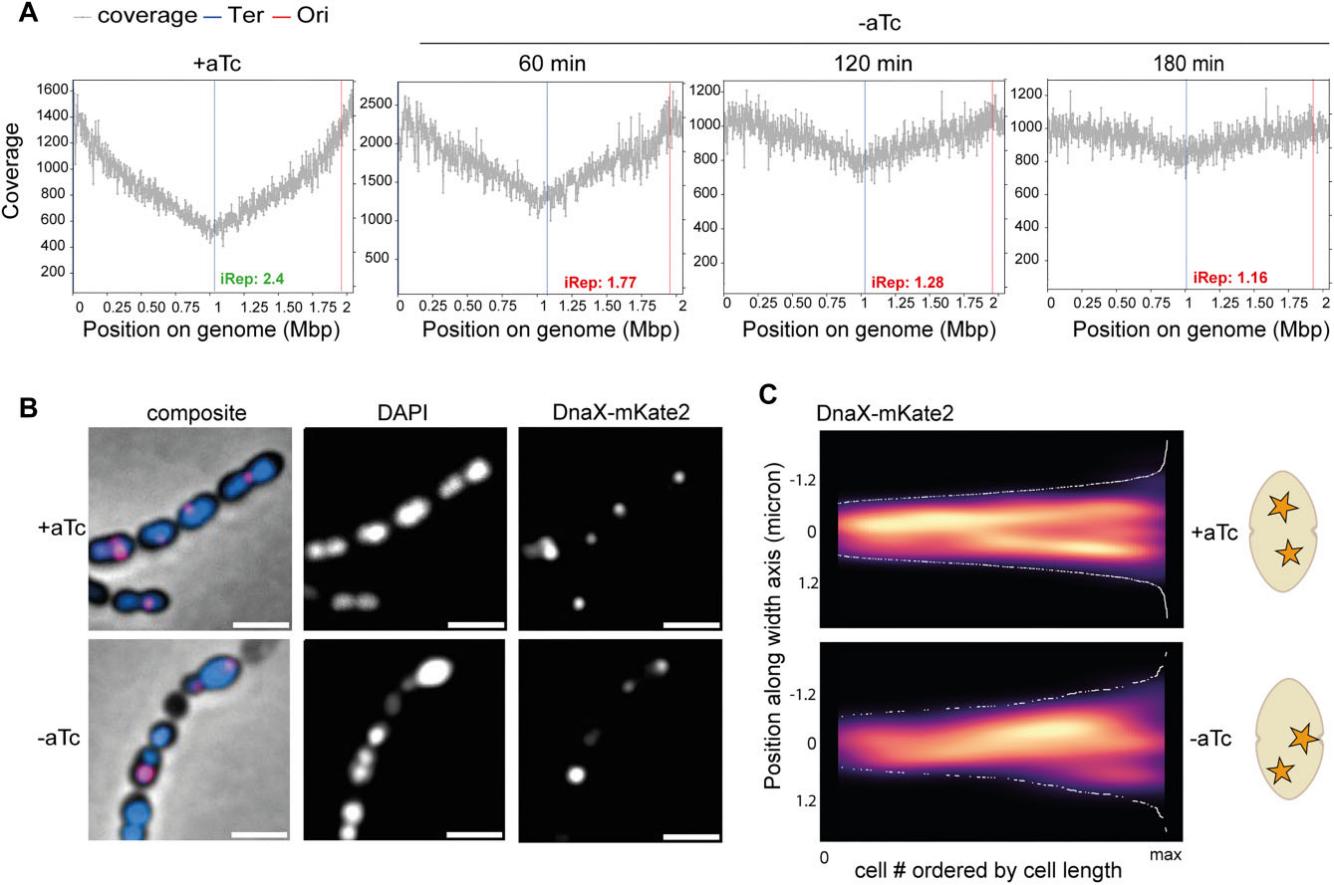
HU-depleted cells progressively stop replication. (**A**) Genome-wide marker frequency analysis. Sequencing reads coverage over the genome sequence of exponentially growing cells in the presence of aTc (on the left) and at one, two, and three h of depletion. The replication rate of the population is indicated by the ratio of coverage at the origin (‘peak’) to the terminus (‘trough’) of replication. The origin and terminus position are determined based on cumulative GC skew. (**B**) DnaX-mKate2 localization in cells depleted for HU (−aTc) for 180 min and non-depleted cells (+aTc). Left to right: composite (phase: grey, DAPI: blue, DnaX-mKate2: pink), DAPI, DnaX-mKate2. (**C**) Kymograph (left) with graphical representation (right). In cells with HU, DnaX is localized at mid-cell and migrates towards the new septa during growth (+aTc, total cells = 313). In cells depleted for HU for 180 min (−aTc, total cells = 705), DnaX is mislocalized.

To investigate whether the lack of HU directly impaired DNA replication, we calculated a iRep based on the sequencing coverage trend of exponentially growing cells and used it to infer replication rates. Cells were grown for 3 h without aTc (HU-depleted cells) and 3 h with the inducer. Genomic DNA was extracted, processed, sequenced, and analysed using iRep [46]. For cells grown in the presence of aTc, reads mapped to the wild-type genome (D39V) and plotted over the correspondent chromosomal position showed a typical profile of exponential growth, with an iRep value (normalized ratio of *ori-*to*-ter* coverage) of 2.4 (Fig. 6A). This value is very similar to that of the D39V wild-type strain [28], indicating that the HU-depletion strain grown in presence of the inducer replicates at a similar rate. Upon HU depletion, the profile became flat, and the iRep value dropped to 1.77 at 1 h, 1.28 at 2 h, and 1.17 at 3 h, indicating that the HU-depleted cells progressively stopped replication.

To follow the DNA replication machine (replisome) in live cells, we fused the clamp loader DnaX with a red fluorescent protein (strain *dnaX::dnaX-mKate2*) and imaged the dynamics of the replisome by fluorescence microscopy (Fig. 6B). Fluorescence microscopy showed enriched signals as bright diffraction-limited spots in the HU-depletion strain grown in presence and absence of the inducer, indicative of active replication. Interestingly, HU depletion decreased the percentage of actively, normally replicating cells (defined as cells with 1–2 DnaX foci) from 66% to 49% (Supplementary Fig. S6A). In wild-type cells, replication forks localise at mid-cell and migrate towards the new septa during growth, while in the absence of HU they are often mislocalized (Fig. 6C), reminiscent of cells with impaired chromosome integrity [36]. Together, these results show that, in the absence of HU, cells fail to initiate replication rounds. When the DNA replication machinery is assembled, further initiation, localization, and progression of the replication fork is impaired.

### HU is genetically intertwined with essential DNA processes

**Figure 7.**
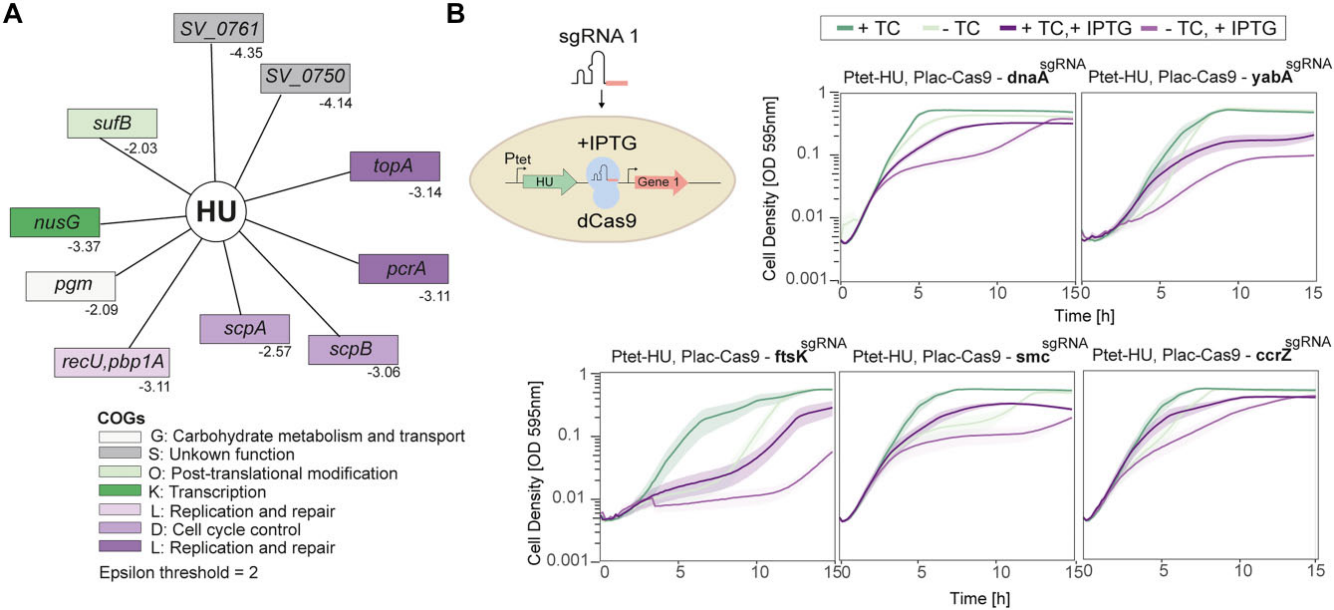
HU is a major structural component essential for chromosome conformation, DNA replication, and chromosome segregation. (**A**) Genetic interaction network of HU as determined by dual-CRISPRi-Seq [76]. Epsilon values are indicated, and a threshold of 2 is set and defined as the added fitness effect of the knock-out of two genes on top of the fitness effects caused by the knock-out of those same two genes individually. (**B**) Growth of knock-down strains obtained by CRISPRi for the DnaA, YabA, CcrZ, and SMC genes in an HU-depletion background. Absence of aTc leads to HU depletion, while presence of IPTG leads to expression of Cas9 and targeted knock-down of each gene.

To gain further insights into the cellular processes to which HU contributes, we exploited the recently generated dual-CRISPRi-seq dataset PneumoGin [76]. By simultaneously downregulating the expression of two genes or operons, a dual-single guide RNA (sgRNA) CRISPRi library was used to screen for genetic interactions in *S. pneumoniae*. These interactions can be negative (simultaneous repression of two genes worsens the growth defect) or positive (simultaneous repression restores growth). By setting a threshold of epsilon of 2, 10 genes or operons are found to be synthetic lethal with HU (Fig. 7A, data available on pneumoGIN). Most of the genes are involved in replication and repair (COG:L) and cell cycle control (COG:D) pathways. These include the DNA topoisomerase I (*topA)*, the helicase *pcrA* and the resolvase *recU*. Proteins required for the positioning of the replication machinery and chromosome segregation, such as *scpA*/*B*, the integral components of the Smc/ScpAB complex, are also synthetic lethal with HU. In addition, knock-down of genes involved in cell size determination (*pgm*) and cell wall biosynthesis (*pbp1a*) worsens an HU phenotype, in line with the deleterious morphology perturbations observed upon HU depletion. Genes with unknown functions (SPV_0750 and SPV_0761) are also part of the HU interaction network, highlighting that HU essentiality might be linked to yet unexplored cellular functions. No positive interactions were identified, indicating that the defects of reduced HU levels cannot be restored by the knock-down of any pneumococcal gene. To investigate the synthetic lethality of genes involved in DNA replication (DnaA, YabA, and CcrZ) and chromosome segregation (SMC and FtsK), we constructed CRISPRi knock-downs and measured their growth (Fig. 7B). To do so, we inserted an IPTG-inducible dCas9 and sgRNA targeting each gene of interest in the Ptet–HU depletion strain. Notably, when growing the cells in the absence of HU (−aTc) and inducing dCas9 expression (+IPTG), we confirmed the synthetic lethality for all genes tested. These interactions enabled us to outline a model of pneumococcal chromosome organization and segregation, incorporating all yet identified partners. In wild-type cells, the nucleoid alternates dynamically between ‘pull’ and ‘twist’ morphologies. The initiator (DnaA) and inhibitor (YabA) of DNA replication, the former controlled by CcrZ, determine the timing of replication and replisome progression. ParB binds to *parS* sites and recruits the SMC complex, which together with FtsK and RocS, segregates the newly replicated chromosomes. In the absence of HU, the supercoiling state of the cell is perturbed, as well as the high-order chromosome folding and the origin of replication. As a result, cells will either have a condensed nucleoid in a larger cell or lose it, either way leading to cell death. In this model, HU plays a central role in maintaining the supercoiled state and building the conformation required for initiation and progression of DNA replication and chromosome segregation.

## Discussion

Most diderm bacteria carry multiple NAPs, whose similar structural properties yield functional redundancy [77]. In contrast, the NAP content of monoderm bacteria has been poorly explored. Although sequence homologues of the IHF, H-NS, or FIS families are absent in the pneumococcus, functional homologues may exist, as observed in other bacterial species [78]. Furthermore, distinguishing between NAPs and transcription factors can be challenging, as both may share DNA-binding properties and serve both structural and regulatory roles [79]. The essentiality of HU in pneumococcus makes this bacterium a particularly interesting model organism for studying its function. Furthermore, due to its high expression [80] and overlap with the nucleoid, HU serves as an ideal marker for tracking the dynamics of the pneumococcal nucleoid. In this study, PALM experiments revealed an extremely compact nucleoid, with HU constantly moving along the chromosome (Fig. 1). Reduced levels of HU resulted in pleiotropic phenotypes, severely impairing cell shape and nucleoid maintenance. After 3 h of depletion, more than one in two cells lost the nucleoid. This strong segregation defect partly answers one of the long-standing questions about how the pneumococcus manages to segregate its chromosome while most of the known factors (SMC, ParB, and FtsK) can be eliminated with only mild phenotypes [35, 36] and places it as an essential factor for successful segregation.

Pneumococcal HU is required to maintain the supercoiling state and counteracts the effects of a less active gyrase [6]. We showed that inhibiting gyrase and topoisomerase IV with a fluoroquinolone resulted in a nucleoid-to-cell ratio that mimicked HU depletion, supporting a critical role of HU in supercoiling, a primary reason for its essentiality. In the absence of HU, the expression of topoisomerase I, IV, and gyrase remains unchanged, suggesting that HU directly impacts supercoiling [6], possibly through the formation of flexible DNA bends.

Although cells lacking HU do not replicate, HU is not required for the assembly of the replication complex. Given the denser binding of HU in proximity of the replication origin and the structural perturbations observed in an HU-depletion strain, we suggest that both supercoiling and architectural defects are responsible for the replication failure. This is also supported by the mislocalization of the replication fork that we measured in the absence of HU.

Hi-C data showed that HU-depleted cells also have lost the interarm chromosome interaction that is characteristic of SMC-mediated chromosome segregation [71–74, 75]. A more precise view of the timing of the consequences of HU depletion could be obtained by repeating these measurements at shorter time points after removal of the inducer or by synchronizing cells and performing single-cell measurements. We, however, speculate that the arrest of DNA replication and DNA segregation occur concomitantly since SMC is not known to require active replication to process DNA. While the impact of HU deletion on chromosome conformation varies among bacterial species [5], the decrease in long-range contacts that we observed has also been noted in *E. coli* and is similar to what is observed in the absence of MukBEF (the *E. coli* condensin), suggesting a cooperation between condensin complexes and HU to promote the higher order organization of the chromosome [Lioy *et al.* (2018)]. This interplay has also been identified in *Streptomyces venezuelae* [81], and we suggest that it could also be the case for *B. subtilis*. In support of this, we find a strong negative genetic interaction between HU and the SMC complex by dual CRISPRi-seq (Fig. 6). We speculate that the hairpin observed in proximity of the *ori*, encompassing one *parS* site, could be explicative of the early loading of ParB/SMC [73, 82] and a failure in proceeding through the chromosomal arms leading to a successful segregation.

Whether the role of HU in DNA replication and segregation involves direct interaction and modulation of DnaA and/or SMC/ParB activity, or their transcriptional regulation, is yet to be elucidated. However, the complementation obtained with *E. coli* HU (59% sequence homology) suggests that unlikely all interactions with homologous proteins are correctly maintained. This rather suggests a structural role required to support replication and the initial steps of loop formation by ParB/SMC. However, to investigate this further, biochemical and structural studies are needed to examine HU interaction partners and binding at *ori*.

Dual-CRISPRi-Seq identified the genetic partners of HU at the genome-wide level [76]. While we cannot exclude the possibility that, given the partial down-expression of HU, other important genetic interactions may be missing, this work has provided a comprehensive view of the cellular pathways to which HU is integrated. Although multiple replication and partitioning factors are responsible for cell cycle progression, HU is strictly required for higher order chromosome conformation and thus DNA replication and chromosome segregation.

## Supplementary Material

gkaf312_Supplemental_Files

## Data Availability

Sequencing data from ChIP-Seq, Hi-C experiment, and Illumina whole genome sequencing are available in the Sequence Read Archive under BioProject accession code PRJNA1169780. ChIP-Seq data obtained for HU-GFP and RpoB-GFP, as well as Hi-C contact matrices, are available for visualization in PneumoBrowse 2.0 [83]. R scripts used for image analysis are made available on GitHub (https://github.com/veeninglab/HUproject) and Figshare (https://doi.org/10.6084/m9.figshare.c.7486716.v1).
