# Supplementary material for: HU promotes higher order chromosome organization and influences DNA replication rates in Streptococcus pneumoniae
Source: Nucleic Acids Res. 2025 Apr 22;53(8):gkaf312. doi: 10.1093/nar/gkaf312 (PMC12014288; doi:10.1093/nar/gkaf312)
Supplement: gkaf312_Supplemental_Files [file gkaf312_supplemental_files.zip › Supplementary Figures.pdf]

Supplementary Figures

Supplementary Figure 1.

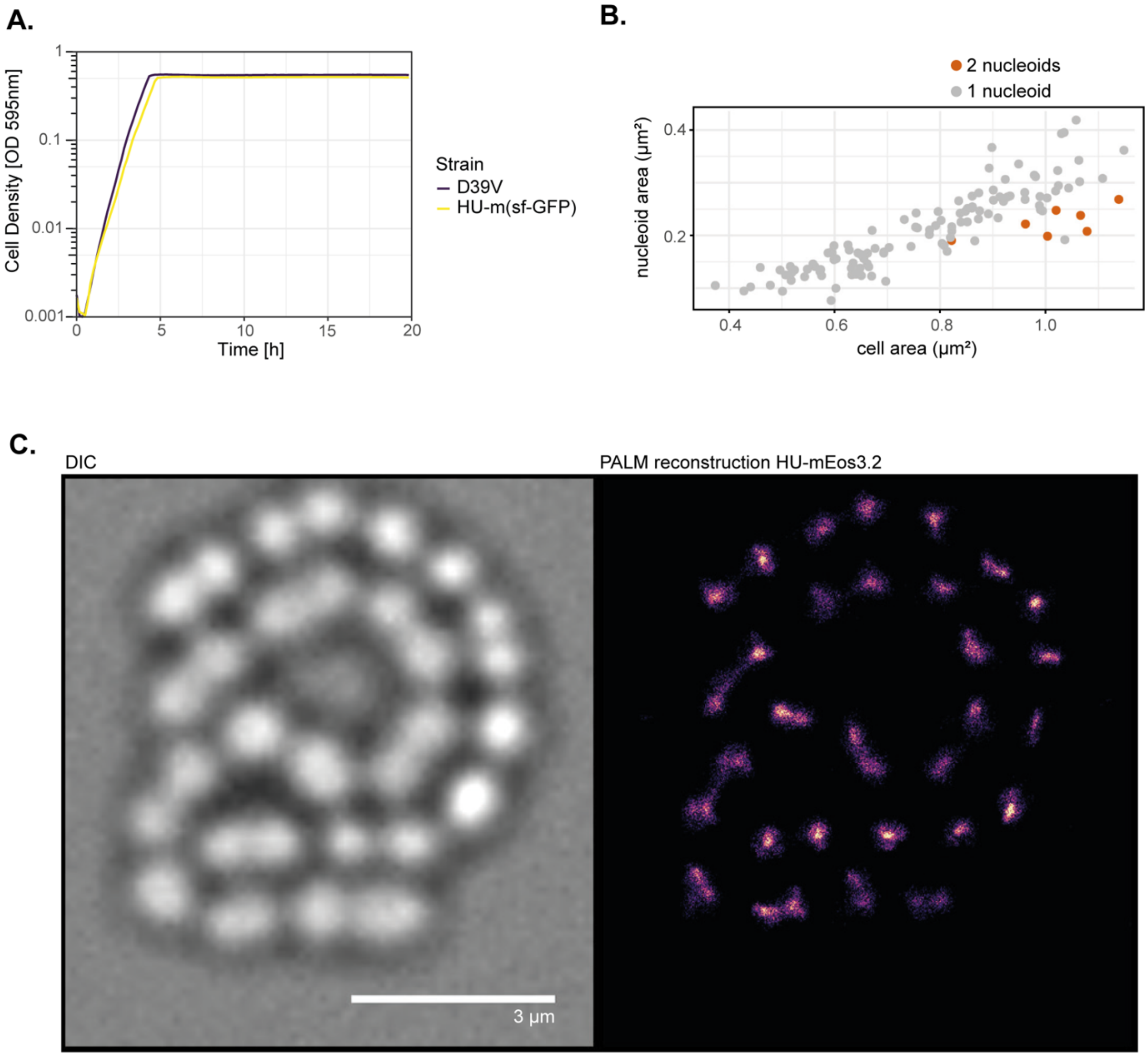

Supplementary figure 1: A. Growth of the strain expressing a functional HU-sfGFP fusion and used for SIM imaging. **B.** Nucleoid to cell area (NC) of cells carrying a functional fusion of HU to superfolder GFP (HU-sfGFP) measured from SIM images. When there are two nucleoids present in one cell, the total nucleoid area is taken (orange data points). **C.** DIC image and corresponding PALM reconstruction of WT D39V carrying *HU-mEos3.2*. Scale bar = 3 micron.

Supplementary Figure 2.

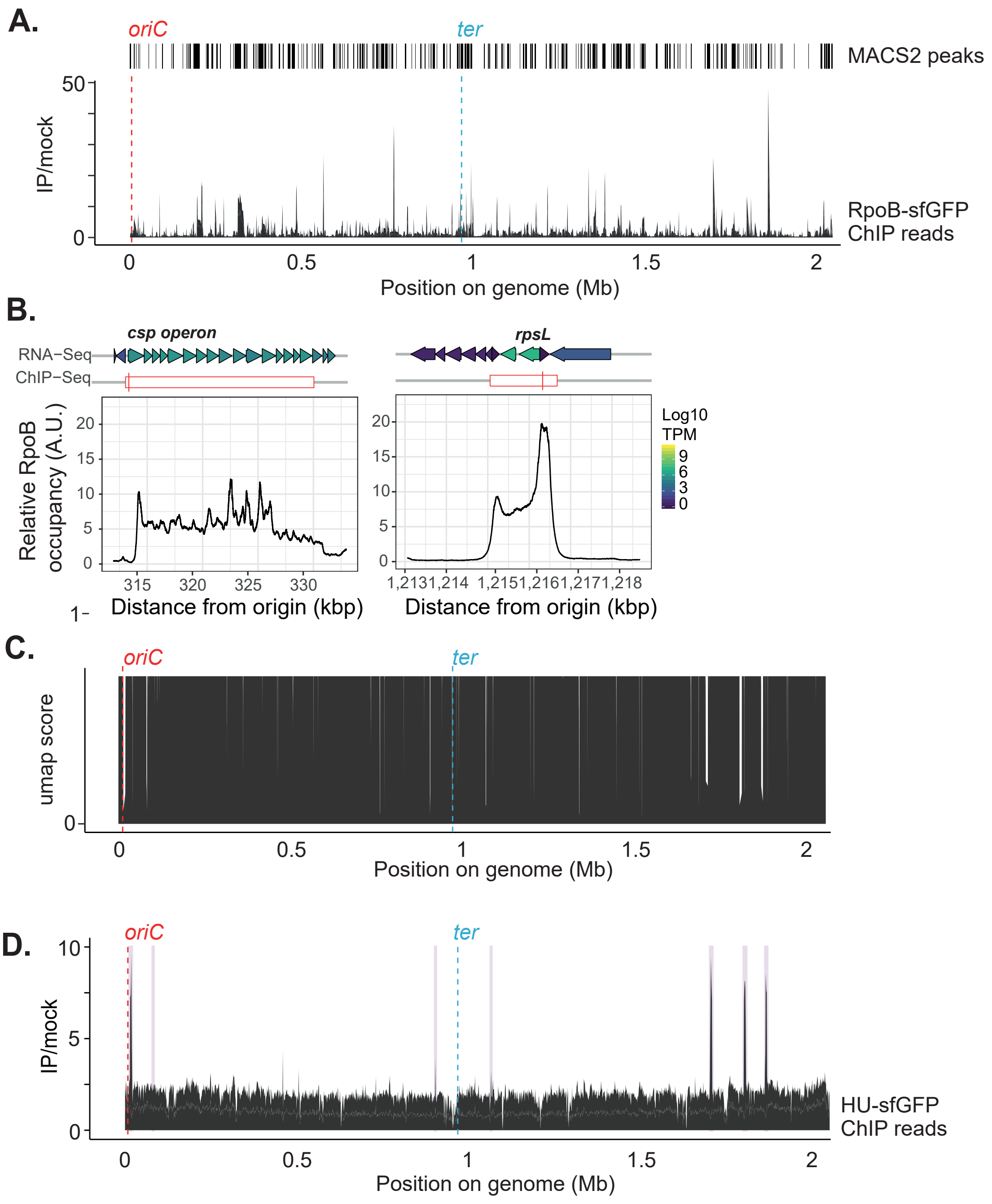

Supplementary Figure 2: A. RpoB relative normalized IP reads over mock (per 1000 bp), where low mappability regions are blacklisted before visualization. Top: RpoB occupation peaks as detected by MACS2. Origin of replication indicated in red, replication terminus in blue. **B.** RpoB occupancy corresponds to elevated expression levels. Top: operon, arrows indicate separate genes, fill indicates expression (log10 TPM, data from Aprianto et al., 2018). Mid: MACS2 detected peaks. Vertical line indicates peak summit. Below: display of relative RpoB occupancy. **C.** Results of mappability analysis of D39V genome using umap. Regions with low mappability (<1) that are larger than 1 kb are excluded from further analysis. Origin of replication indicated in red, replication terminus in blue. **D.** HU-sfGFP normalized IP reads over mock (per 1000 kb) before blacklisting shows one large peak close to the

origin and three between 1.5-2 Mb. These regions overlap with the blacklisted regions shown in purple. Origin of replication indicated in red, replication terminus in blue.

Supplementary Figure 3.

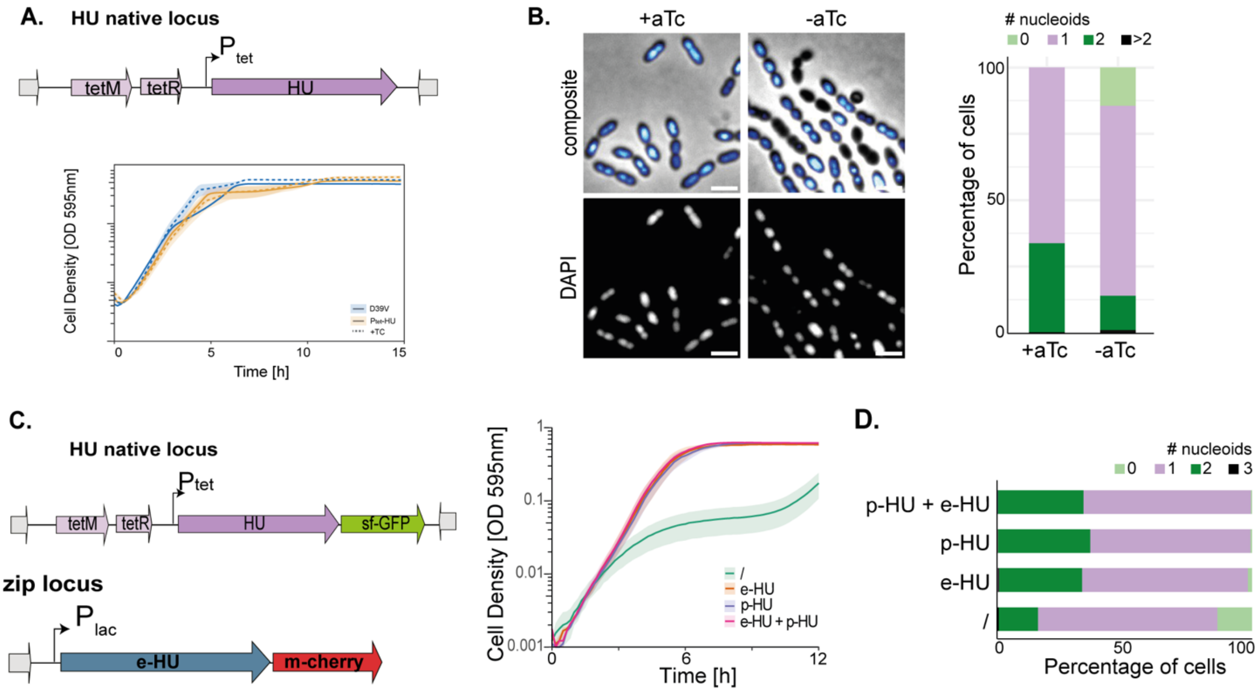

Supplementary figure 3: A. Growth curves of wild-type D39V and Ptet-HU (+/- aTc) strains. **B.** Phase contrast and DAPI epi-fluorescent images and nucleoid measurements of Ptet-HU strain. **C.** Growth curves of Ptet-HU-sfGFP grown in presence and absence of the inducer (+/- aTc) and complemented with *E.coli* HU (e-HU) expressed under an inducible Plac promoter (+/- isopropyl-β-D-thiogalactoside (IPTG)). **D.** Nucleoid measurements for strains lacking HU or expressing either e-HU, p-HU or a combination of both.

Supplementary Figure 4.

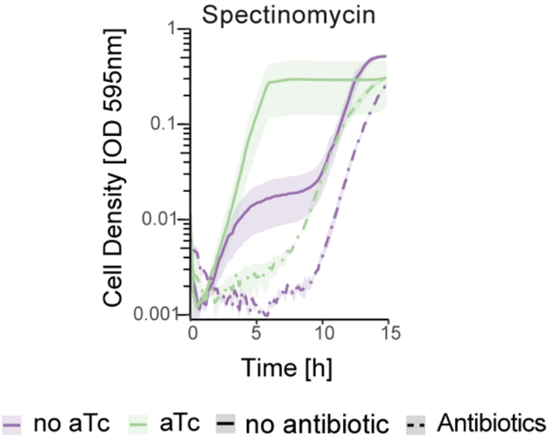

Supplementary figure 4: Growth curves of HU depletion strain (+/- aTc) grown in presence and absence of low doses of spectinomycin (10 µg ml−1).

Supplementary Figure 5.

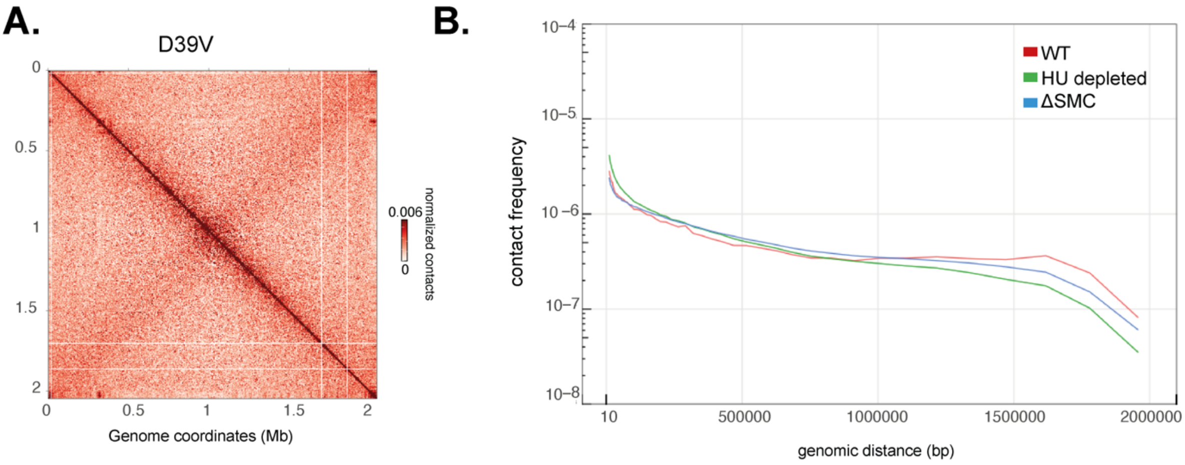

Supplementary figure 5: A. Hi-C map of wild-type strain D39V. **B.** Contact probability curves (p(s)) for WT, HU depleted and Δsmc strains. The curves represent the averaged probability of a contact (y-axis) between genomic loci separated by a given genomic distance indicated in the x-axis.

Supplementary Figure 6.

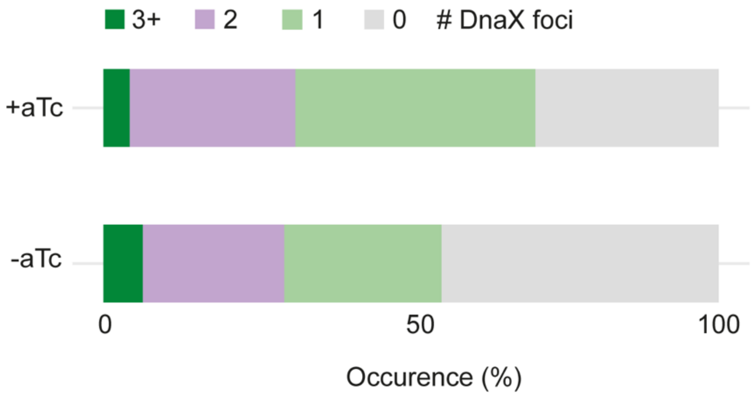

Supplementary figure 6: Number of DnaX-mKate2 foci measured by flurorescence miscroscopy of HU complemented (+aTc) and depleted (-aTc) strain.
